# Primary Bone Lymphoma in Adults: A Report of Two Cases and Review of the Literature Highlighting the Role of Radiotherapy

**DOI:** 10.7759/cureus.106791

**Published:** 2026-04-10

**Authors:** Veronia Fahmy, Anna Huynh, Alec M Block, James Welsh

**Affiliations:** 1 Radiation Oncology, Loyola University Chicago Stritch School of Medicine, Maywood, USA; 2 Oncology, University of Texas Medical Branch at Galveston, Galveston, USA; 3 Radiation Oncology, Edward Hines Jr. Veterans Administration Hospital, Hines, USA

**Keywords:** bone neoplasm, consolidative therapy, diffuse large b-cell lymphoma (dlbcl), extranodal lymphoma, multimodal therapy, pet/ct imaging, primary bone lymphoma, radiation, rare malignancy, r-chop

## Abstract

Primary bone lymphoma (PBL) is a rare extranodal hematologic malignancy that can pose significant diagnostic challenges. Patients commonly present with localized pain, swelling, pathological fracture, or neurological deficits, and the disease may mimic infection, degenerative conditions, trauma, or metastatic carcinoma. Lesions can arise in any skeletal site, underscoring the importance of interdisciplinary awareness among orthopedics, neurosurgery, dentistry, and oncology. Histologically, diffuse large B-cell lymphoma is the most frequently encountered subtype, with certain molecular features carrying important prognostic implications. Accurate diagnosis depends on adequate tissue sampling, immunophenotyping, and staging with positron emission tomography/computed tomography, and repeat or open biopsy may be required in select cases. Management is typically optimized with combined chemo-immunotherapy, most commonly rituximab-based regimens, with or without consolidative radiotherapy to improve local control and symptom relief. Surgical intervention is generally reserved for diagnostic purposes or skeletal stabilization. We report two cases of elderly patients, one Middle Eastern male and one White female, with stage IE PBL involving the sacrum and iliac crest, respectively. Both patients were treated with systemic therapy followed by salvage involved-site radiotherapy, achieving durable local disease control and symptom resolution. Early recognition of PBL enables effective multimodal treatment and favorable clinical outcomes. This case series highlights the diagnostic complexity of PBL, emphasizes the role of radiotherapy in management, and underscores the need for standardized treatment approaches to optimize care for this rare lymphoma subtype.

## Introduction

Primary bone lymphoma (PBL) is a rare malignant hematologic neoplasm that accounts for a small proportion of all lymphomas and extranodal lymphoid malignancies. The most common histologic subtype is diffuse large B-cell lymphoma (DLBCL), and PBL represents approximately 2% of all primary bone tumors and 5% of extranodal lymphomas [1]. PBL is defined as a lymphoma arising within the medullary cavity of bone, with or without limited regional lymph node involvement, and without evidence of disseminated nodal or visceral disease at the time of diagnosis [2]. Distinguishing PBL from secondary osseous involvement by systemic lymphoma is clinically important, as primary bone disease is associated with more favorable outcomes compared with secondary skeletal involvement [3].

Histologically, DLBCL predominates among cases of PBL, while other subtypes, including follicular lymphoma, T-cell lymphoma, and rare molecular variants, are reported less frequently [4]. The femur is the most commonly affected site, followed by other long bones, the pelvis, spine, skull, and, less commonly, the scapula or craniofacial bones [2]. Clinical presentation is often nonspecific and may include localized bone pain, swelling, pathological fracture, or neurological symptoms when lesions involve the spine or skull base [5,6]. Radiographic findings range from subtle lytic or sclerotic changes to aggressive bone destruction with associated soft-tissue mass, frequently necessitating further evaluation with computed tomography (CT), magnetic resonance imaging (MRI), or positron emission tomography/computed tomography (PET/CT) for accurate staging and treatment planning [7].

Definitive diagnosis relies on histopathological evaluation and immunohistochemical analysis obtained through biopsy, allowing differentiation from other primary bone tumors and metastatic disease [1,3]. Although PBL generally carries a more favorable prognosis than other primary malignant bone tumors or secondary skeletal lymphoma, standardized treatment algorithms remain lacking due to the rarity of the disease [5]. Available evidence supports a combined-modality treatment approach using chemo-immunotherapy, often with consolidative radiotherapy, with most data derived from retrospective series, systematic reviews, and individual case reports [5,8].

However, important clinical challenges remain insufficiently characterized in the literature, particularly in patients with atypical anatomic presentations, inconclusive initial diagnostic studies, or variable responses to systemic therapy requiring treatment adaptation. These issues are especially relevant in cases involving the axial skeleton or pelvis, where symptoms may be misleading and management decisions, including radiotherapy dose selection, are less clearly defined.

In this report, we present two cases of stage IE primary bone DLBCL involving the sacrum and iliac crest, respectively. These cases highlight diagnostic complexity, including initially non-diagnostic findings and confounding incidental lesions, as well as the need for individualized, response-adapted treatment strategies. In particular, we emphasize the role of radiotherapy in achieving durable local control, including the use of standard versus escalated dosing in the setting of residual disease.

## Case presentation

Case 1: Primary sacral DLBCL in a 64-year-old Middle Eastern male

Chief Complaint

A 64-year-old Middle Eastern male presented with left lower extremity pain and difficulty initiating urination.

History of Present Illness

The patient initially experienced left lower extremity discomfort in December 2024. In February 2025, he developed progressive difficulty initiating urination. These urinary symptoms were concerning for sacral nerve root involvement secondary to mass effect from the lesion. MRI of the sacrum revealed a mass involving the entire sacrum, with abnormal marrow signal intensity extending from S1 through S5. The lesion demonstrated contrast enhancement and was associated with a presacral soft-tissue mass measuring 5.1 × 3.4 cm in the axial plane, predominantly left of midline. The mass exhibited an infiltrative pattern, involving the left sacroiliac joint and causing compromise of the sacral spinal canal, with additional involvement of multiple sacral neural foramina.

CT of the chest, abdomen, and pelvis demonstrated the presacral mass with underlying sacral bone resorption and a nondisplaced pathological fracture, consistent with osseous involvement by lymphoma. No additional osseous or visceral lesions were identified. MRI of the brain with and without contrast showed no evidence of metastatic disease.

The patient denied constitutional B symptoms, including fever, night sweats, or unintentional weight loss. He had no significant past medical history and no prior surgical history. He reported no known drug allergies and was not taking any medications. Family history was noncontributory. He denied tobacco use, alcohol consumption, and recreational drug use.

Physical Examination

Physical examination was notable for tenderness to palpation over the sacrum. No focal neurologic deficits were identified.

Laboratory Findings

Flow cytometry of the biopsy specimen demonstrated a mixed population of lymphoid cells, including CD19-positive B cells and CD3-positive T cells. The B-cell population showed polyclonal expression of kappa and lambda light chains, suggesting a reactive rather than clonal process. These findings were initially non-diagnostic and contributed to diagnostic uncertainty, necessitating definitive histopathologic evaluation. Fluorescence in situ hybridization (FISH) testing was negative for cytogenetic abnormalities. Lactate dehydrogenase (LDH) levels were within normal limits.

Imaging Findings

MRI of the sacrum demonstrated diffuse replacement of normal marrow signal from S1 through S5, with an associated presacral soft-tissue mass measuring 5.1 × 3.4 cm, involvement of the left sacroiliac joint, and compromise of the sacral spinal canal. CT of the chest, abdomen, and pelvis confirmed the presacral mass with sacral bone resorption and a nondisplaced pathological fracture, without evidence of additional lesions. MRI of the brain was negative for intracranial or leptomeningeal disease.

Pathology and Final Diagnosis

Histopathologic examination of the sacral biopsy confirmed DLBCL, a germinal center B-cell subtype, primarily involving bone, consistent with PBL. The disease was classified as bulky stage IE with a low-risk International Prognostic Index (IPI) based on age.

Treatment and Clinical Course

The patient completed six cycles of rituximab, cyclophosphamide, doxorubicin, vincristine, and prednisone (R-CHOP) chemotherapy. A post-treatment PET/CT scan performed four months after completion of chemotherapy demonstrated a complete metabolic response, with only a faint residual fracture line in the left sacrum. A follow-up CT scan obtained one month later showed interval reduction in presacral soft-tissue thickening compared with pre-chemotherapy imaging.

**Figure 1:**
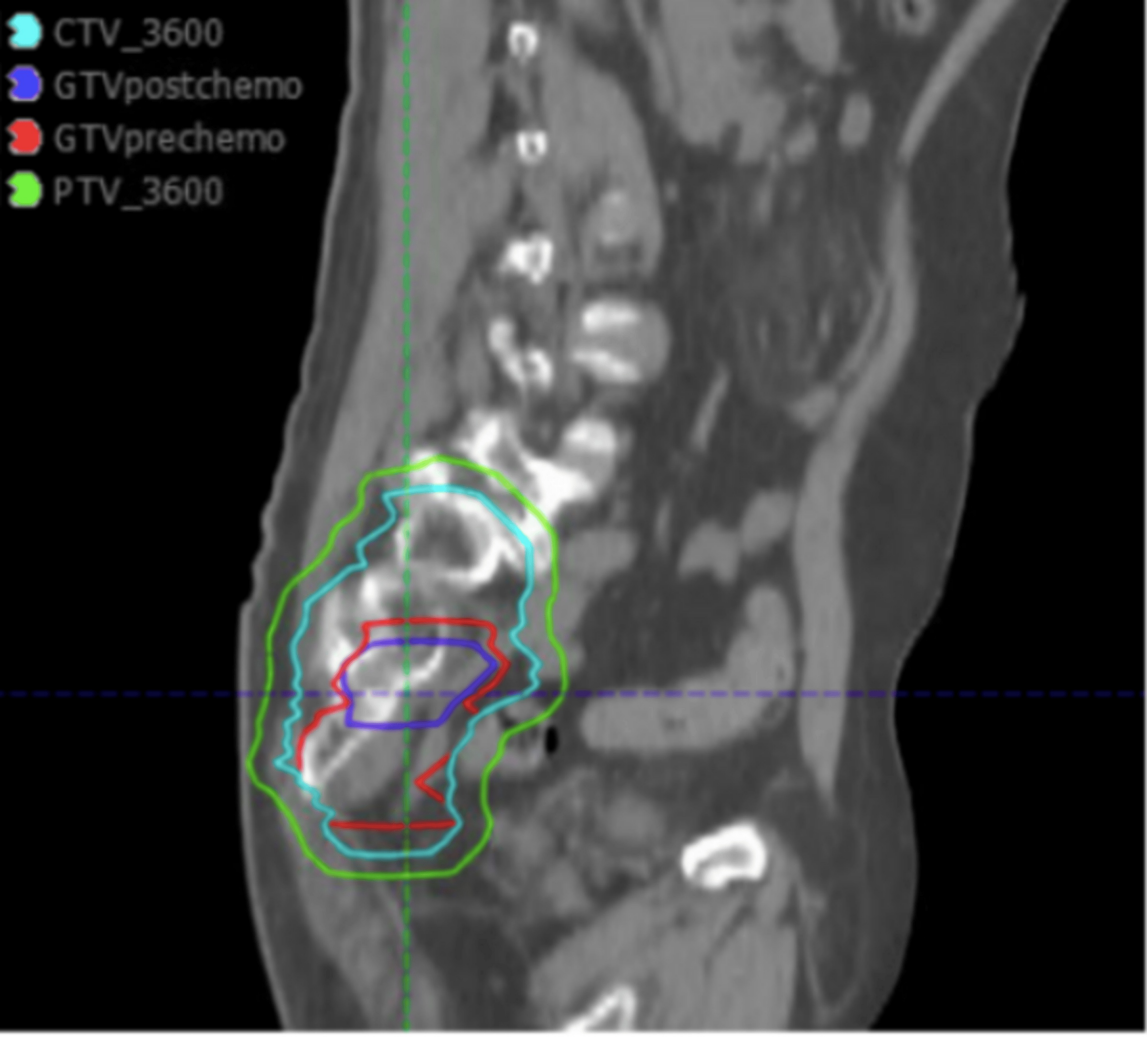
Target volume delineation before and after chemotherapy. Target volume delineation for the presented case. The gross tumor volume before chemotherapy (GTV pre-chemotherapy) and after chemotherapy (GTV post-chemotherapy) is shown. These volumes were expanded to generate the clinical target volume receiving 36.00 Gy (CTV_3600), followed by the addition of a uniform margin to create the planning target volume receiving 36.00 Gy (PTV_3600).

The patient subsequently underwent consolidative involved-site radiotherapy to the left sacrum without inclusion of regional lymph nodes. As shown in Figure 1, radiation target volume delineation incorporated the pre-chemotherapy gross tumor volume and the residual post-chemotherapy gross tumor volume, which were expanded to generate the clinical target volume receiving 36 Gy (CTV_3600). A uniform margin was applied to create the planning target volume receiving 36 Gy (PTV_3600). The total prescribed dose was 36 Gy delivered in 18 daily fractions, administered Monday through Friday.

Case 2: Right gluteal and iliac crest DLBCL in a 72-year-old White female

Chief Complaint

A 72-year-old White female presented after a mechanical fall resulting in a lumbar vertebral fracture.

History of Present Illness

The patient sustained a fall on May 7, 2024, and presented to the emergency department. CT of the head was unremarkable, while CT of the spine demonstrated an L3 vertebral fracture. Incidental findings included a right thyroid mass, a small left adrenal nodule, and apical lung nodules. These findings were further evaluated and determined not to represent disseminated lymphoma. The thyroid lesion was ultimately diagnosed as a separate follicular neoplasm, while the remaining findings were considered clinically insignificant or unrelated. Subsequent PET/CT performed on June 20, 2024, revealed a hypermetabolic right gluteal soft tissue mass measuring 5.5 × 6.5 cm with a maximum standardized uptake value (SUV) of 36. Additionally, a hypermetabolic right lower neck and thyroid mass measuring 4.5 × 4.5 cm was identified, extending into the mediastinum with associated tracheal narrowing. A left adrenal nodule measuring 0.9 cm (SUV = 4.3) and small apical lung nodules were also noted.

A core needle biopsy of the right gluteal mass performed on July 12, 2024, confirmed DLBCL, non-germinal center B-cell subtype. The patient denied constitutional B symptoms, including fever, night sweats, or unintentional weight loss. She did report weight loss that she attributed to decreased oral intake secondary to pain and functional limitations. Family history was notable for breast cancer in her mother and sister. She denied tobacco and alcohol use.

Physical Examination

Physical examination revealed tenderness over the right gluteal region and right iliac crest. No focal neurologic deficits were present.

Laboratory Findings

Bone marrow biopsy demonstrated normocellular marrow (25-30%) without evidence of lymphoma involvement. Cytogenetic analysis revealed trisomy 12. LDH levels were persistently elevated. Thyroid function testing, including thyroid-stimulating hormone (TSH) and free thyroxine (T4), was within normal limits.

Imaging Findings

**Figure 2:**
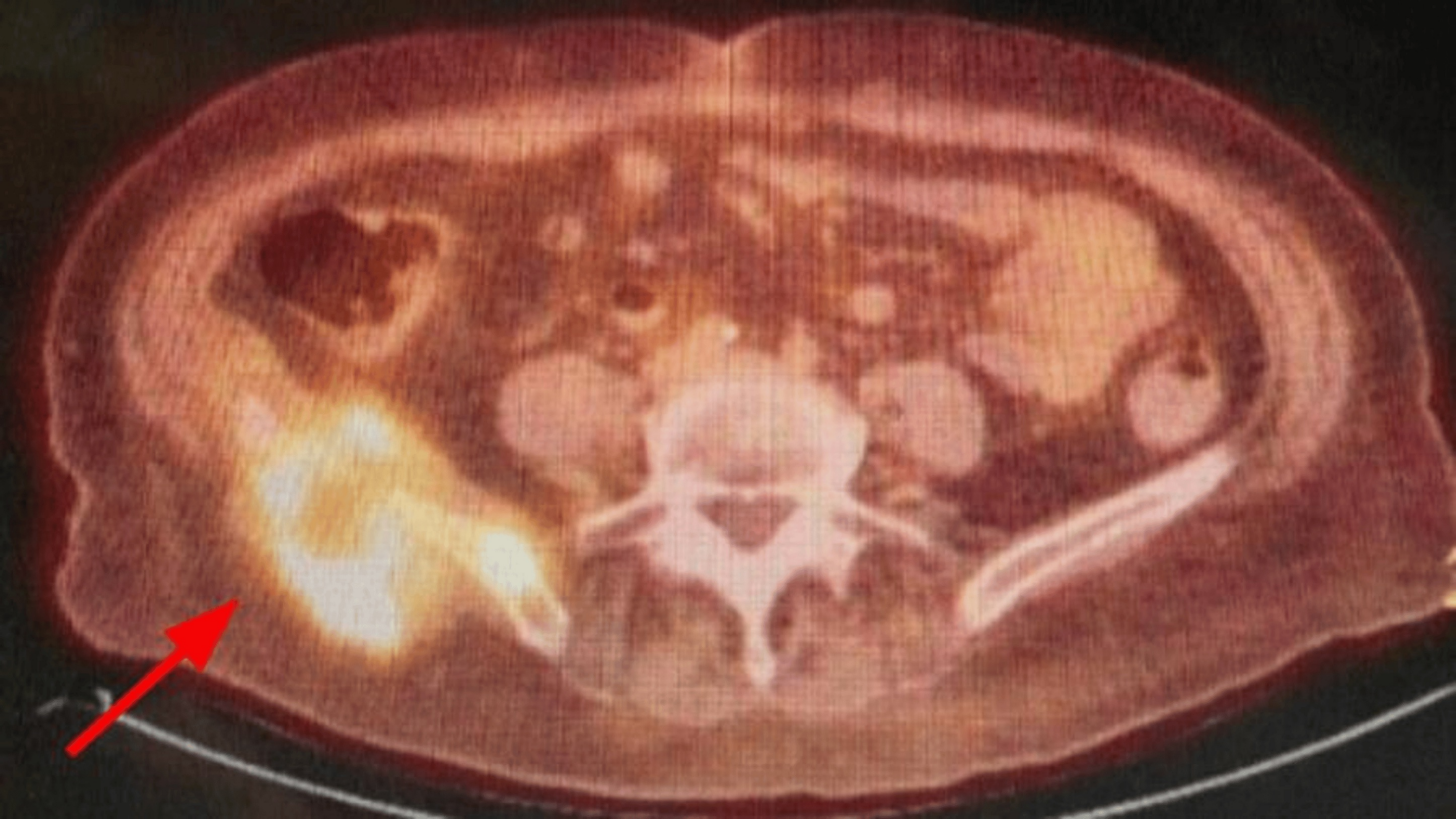
Fluorodeoxyglucose uptake at the right gluteal crest on positron emission tomography/computed tomography.

CT imaging of the spine confirmed an L3 vertebral fracture and identified an incidental right thyroid mass. PET/CT demonstrated a hypermetabolic right gluteal soft tissue mass measuring 5.5 × 6.5 cm (SUV = 36), a hypermetabolic right thyroid mass measuring 4.5 × 4.5 cm with mediastinal extension and tracheal compression, and mild fluorodeoxyglucose (FDG) uptake associated with cortical irregularity and osseous destruction of the adjacent right iliac crest, as shown in Figure 2.

MRI of the pelvis further characterized the right gluteal mass, measuring 9.6 × 5.8 × 6.3 cm. Follow-up PET/CT imaging after systemic therapy demonstrated marked interval improvement of the gluteal mass, with residual mild metabolic activity and persistent cortical irregularity of the right iliac bone.

Pathology and Final Diagnosis

Histopathologic analysis of the right gluteal mass demonstrated DLBCL, non-germinal center B-cell subtype, composed of large atypical lymphoid cells positive for CD20, B-cell lymphoma 2 (BCL2), B-cell lymphoma 6 (BCL6), MYC, and CD30 in a subset of cells. The Ki-67 proliferation index was approximately 70%. FISH revealed a BCL6 rearrangement, while MYC and BCL2 rearrangements were negative. Bone marrow biopsy was negative for lymphoma involvement. The findings were consistent with stage IE PBL involving the right iliac crest, classified as low-intermediate risk by the IPI based on age and persistently elevated LDH. The thyroid mass was diagnosed as a separate follicular neoplasm.

Treatment and Clinical Course

The patient initially received one cycle of R-CHOP, followed by five cycles of polatuzumab vedotin with rituximab, cyclophosphamide, doxorubicin, and prednisone (Pola-R-CHP), due to concern for suboptimal response and higher-risk disease features, including non-germinal center subtype and elevated LDH. Given residual metabolic activity on follow-up imaging, salvage systemic therapy with obinutuzumab and rituximab was administered for six cycles to improve disease control.

Following systemic therapy, the patient underwent consolidative radiotherapy to the right gluteal and iliac region, receiving a total dose of 50 Gy in 25 fractions. The decision to escalate radiation dose beyond standard ranges was based on persistent metabolic activity after multiple lines of systemic therapy, raising concern for residual or refractory disease requiring intensified local treatment.

Chemotherapy was generally well tolerated; the patient developed peripheral neuropathy, which stabilized over time without progression.

## Discussion

PBL is an uncommon malignancy, and most available data derive from small retrospective series and case reports [9]. As a result, variability exists in diagnostic approaches, treatment strategies, and reporting of outcomes, particularly in atypical presentations and complex clinical scenarios [10].

Across published reports, PBL most frequently manifests as a localized form of DLBCL, which constitutes the predominant histologic subtype in the majority of adult cases [1]. Less common variants, including non-germinal center subtypes and rare molecularly defined entities, have also been described but remain exceptional [9].

Clinically, patients often present with localized bone pain, swelling, or pathological fracture, reflecting the destructive behavior of the disease within the medullary cavity [9]. Neurological symptoms may arise when lesions involve the spine, skull base, or cranial vault, occasionally resulting in nerve palsies or spinal cord compression [11].

PBL can affect both the appendicular and axial skeleton. The femur is the most frequently involved site, followed by other long bones such as the tibia and humerus, as well as the pelvis, scapula, and vertebral column [12]. Craniofacial involvement, including the cranial vault, skull base, temporal bone, mandible, and maxilla, has also been reported, often presenting diagnostic and therapeutic challenges due to proximity to critical neurovascular structures [6].

Several reports describe an indolent clinical course with prolonged nonspecific or intermittent pain before diagnosis, particularly in lesions involving the scapula, long bones, or pelvis [12]. In contrast, other cases demonstrate atypical or misleading presentations, including soft-tissue masses, cutaneous extension, or systemic manifestations mimicking inflammatory or degenerative conditions [13].

This heterogeneity highlights the diagnostic difficulty of PBL and underscores the importance of maintaining a high index of suspicion in patients with persistent focal bone pain, unexplained destructive bone lesions, or atypical skeletal masses, especially when imaging findings are inconclusive [11]. Such clinical and anatomic variability also has important implications for radiotherapy planning, including target volume delineation, dose selection, and integration with systemic therapy in combined-modality treatment [6].

Treatment regimens for adult PBL, including chemotherapy and consolidative radiotherapy, are summarized in Table 1. Most reported cases utilized R-CHOP- or CHOP-based regimens, cyclophosphamide, doxorubicin hydrochloride, vincristine sulfate, rituximab, and prednisone, often combined with involved-field or involved-site radiotherapy [12].

**Table 1:** Adult primary bone lymphoma: summary of cases and treatment with chemotherapy and radiotherapy. Dose and fractionation are reported where available; NR indicates not reported in the original publication. DLBCL = diffuse large B-cell lymphoma; THRLBCL = T-cell/histiocyte-rich large B-cell lymphoma; NHL = non-Hodgkin lymphoma; R-CHOP = rituximab + cyclophosphamide, doxorubicin, vincristine, prednisone; CHOP = cyclophosphamide, doxorubicin, vincristine, prednisone; M = male; F = female

Author (year)	Histology (N)	Median age (years)	Sex	Site	Ann Arbor stage	Treatment
Scoccianti et al. (2013) [1]	DLBCL 23/27	52	M	Femur, tibia, pelvis, spine	I–IV	CHOP or R-CHOP ± RT^1^
Singh et al. (2010) [9]	DLBCL 2/2	45, 55	M	Long bones	I	CHOP ± RT^1^
Chigurupati et al. (2021) [14]	DLBCL 1/1	52	M/F	Sacrum	I	R-CHOP ± RT^1^
Liu et al. (2014) [5]	DLBCL 1/1	46	M	Radius	I	R-CHOP ×6 ± RT^1^
Cortese et al. (2014) [6]	DLBCL 2/2	50s	M	Maxillofacial bones	I	CHOP-based ± RT^1^
Undabeitia et al. (2014) [15]	DLBCL 1/1	45	M	Cervical spine	I	R-CHOP ± RT^1^
Kiamos et al. (2023) [16]	DLBCL 1/1	60s	M	Femur	I	CHOP ± RT^1^
Giardino et al. (2012) [12]	DLBCL 1/1	59	M	Bilateral tibia	I	CHOP ± RT^1^
Nakazato et al. (2009) [17]	DLBCL 1/1	63	M	Scapula	I	R-CHOP ± RT^1^
Razakanai vo et al. (2015) [18]	NHL (DLBCL presumed) 1/1	Adult	M	Long bone	I	CHOP-based ± RT^1^
Shimada et al. (2013) [2]	DLBCL (MYC+) 1/1	65	M	Sacrum	I	R-CHOP ± RT^1^
Sugimoto et al. (2014) [19]	DLBCL 1/1	55	M	Cranial vault	I	CHOP ± RT^1^
Fadoukhair et al. (2011) [3]	NHL (DLBCL likely) 1/1	42	M	Cranial vault	I	CHOP ± RT^1^
Uchida et al. (2021) [13]	DLBCL 1/1	69	M	Cranial vault	I	R-CHOP ± RT^1^
Guan et al. (2023) [10]	DLBCL 1/1	58	M	Skull base	I	R-CHOP ± RT^1^
Jiang et al. (2011) [11]	NHL (DLBCL suspected) 1/1	62	F	Temporal bone	I	CHOP-based ± RT^1^
Zou et al. (2018) [8]	DLBCL 1/1	67	F	Maxilla	I	R-CHOP ± RT^1^
Buchanan et al. (2015) [7]	DLBCL 1/1	Adult	F	Head and neck bone	I	R-CHOP ± RT^1^
Kini et al. (2009) [4]	DLBCL 1/1	52	M	Mandible	I	CHOP ± RT^1^

DLBCL represented the majority of adult PBL cases summarized in Table 1, with rarer histologies, such as T-cell/histiocyte-rich large B-cell lymphoma, also reported. Patients ranged widely in age, typically during the fifth to seventh decades of life, with a slight male predominance, reflecting the demographic trends shown in the table. The most frequently affected bones included the femur, tibia, pelvis, cranial vault, and mandible, involving both axial and appendicular sites. Most cases were localized (Ann Arbor stage I), although a smaller proportion presented with more advanced disease (stages II-IV). Chemotherapy, predominantly R-CHOP- or CHOP-based regimens, was the mainstay of treatment, with radiotherapy commonly delivered as consolidative therapy in the majority of patients. These data highlight that while chemotherapy remains the backbone of management, radiotherapy is frequently employed in localized disease, supporting its role as consolidative therapy for adult PBL.

Histopathology and molecular features

Histologically, DLBCL predominates in adult PBL, affecting sites such as the cranial vault, long bones, and axial skeleton [3]. Immunophenotyping consistently demonstrates CD20 positivity, with additional markers including Paired Box 5 (PAX5), CD10, and BCL6 reported when available [16]. Proliferative indices vary, ranging from approximately 40% in indolent cases to greater than 80% in aggressive presentations [8]. Molecular profiling identifies non-germinal center B-cell-like (GCB) subtypes and MYC rearrangements, which have been associated with poorer prognosis [2].

Diagnosis and imaging pitfalls

The diagnosis of PBL remains challenging. Early radiographs may appear normal or show nonspecific changes, whereas MRI can mimic infection or other inflammatory processes [16]. FDG-PET/CT is essential for accurate staging and for distinguishing monostotic disease from multifocal or systemic involvement [14]. In some cases, repeated or open biopsies are required for definitive diagnosis, emphasizing the need for adequate tissue sampling and integration of histologic findings with immunophenotyping [5].

Treatment and the role of radiotherapy

In adult PBL, combined chemotherapy and radiotherapy has consistently demonstrated favorable outcomes across multiple series and case reports, as summarized in Table 2. Chemotherapy, predominantly R-CHOP- or CHOP-based regimens, remains the backbone of treatment, while radiotherapy is frequently employed as consolidative therapy, particularly in localized disease. Across published series, combined-modality therapy is associated with excellent outcomes, with local control rates exceeding 85-90% and five-year overall survival approaching 90% in patients with localized disease.

**Table 2:** Radiotherapy characteristics and outcomes in adult primary bone lymphoma. Dose and fractionation are reported where available; NR indicates not reported in the original publication. Dose given in Gray (Gy); fractionation generally conventional 1.8–2 Gy/day unless otherwise specified. Timing refers to when RT was delivered relative to systemic chemotherapy. OS = overall survival; NR = not reported; RT = radiotherapy

Study	Patients (N)	Site(s)	RT dose (Gy)	Fractionation	Timing	Outcomes
Scoccianti et al. (2013) [1]	NR	Various bones	36–40	Fractionated	After chemotherapy	Excellent local control; OS ~90%; surgery only for stabilization
Uchida et al. (2021) [13]	1	Calcaneus	36	Conventional	After chemotherapy	Symptom relief and local control
Kiamos et al. (2023) [16]	1	Femur	40	Conventional	After chemotherapy	Local control achieved; pain resolution
Fadoukhair et al. (2011) [3]	1	Cranial vault	36	Conventional	After chemotherapy	Local control; OS NR
Sugimoto et al. (2014) [19]	1	Skull	40	Conventional	After chemotherapy	Symptom resolution; local disease controlled
Liu et al. (2014) [5]	1	Radius	36	Conventional	After chemotherapy	Complete remission; durable local control
Undabeitia et al. (2014) [15]	1	Cervical spine	36–40	Conventional	After chemotherapy	Local control, neurologic symptom improvement
Cortese et al. (2014) [6]	2	Mandible, maxillofacial soft tissue	36	Conventional	After chemotherapy	Local control maintained; OS NR
Giardino et al. (2012) [12]	1	Bilateral tibia	36	Conventional	After chemotherapy	Pain relief and local disease control
Nakazato et al. (2009) [17]	1	Scapula	36	Conventional	After chemotherapy	Complete remission; functional recovery
Razakanaivo et al. (2015) [18]	1	Femur	36	Conventional	After chemotherapy	Local control; OS NR
Shimada et al. (2013) [2]	1	Sacrum	36	Conventional	After chemotherapy	Complete remission; local disease controlled
Guan et al. (2023) [10]	1	Skull base	36	Conventional	After chemotherapy	Local control; symptom improvement
Zou et al. (2018) [8]	1	Maxilla	36	Conventional	After chemotherapy	Complete response; OS NR
Buchanan et al. (2015) [7]	1	Head and neck	36	Conventional	After chemotherapy	Local control achieved; OS NR

Radiotherapy and site-specific considerations

Radiotherapy is typically delivered after systemic therapy, with doses most commonly in the range of 30-40 Gy using conventional fractionation (1.8-2 Gy per day) [20]. This approach achieves durable local control, particularly in patients with a good response to chemo-immunotherapy. However, higher radiotherapy doses, up to 50 Gy, may be considered in select cases with bulky disease or residual metabolic activity following systemic therapy, where there is concern for persistent or refractory disease, as demonstrated in our second case [20]. Surgery is generally reserved for diagnostic biopsy or structural stabilization rather than for oncologic control [1]. FDG-PET/CT is frequently employed to guide target volume delineation, adapt treatment volumes based on response, and distinguish residual tumor from post-treatment changes [4].

These approaches are consistent with current guideline recommendations, including those from the National Comprehensive Cancer Network and the International Lymphoma Radiation Oncology Group, which support combined-modality therapy and the use of consolidative radiotherapy in localized disease, with dose adaptation based on treatment response and clinical factors.

Case reports highlight the clinical diversity of radiotherapy applications. For example, cranial vault lesions were effectively treated with 36 Gy after chemotherapy [3,19], calcaneal and tibial lesions responded well to 36-40 Gy [13,16], and craniofacial or mandibular disease achieved durable local control with 36 Gy [6,7,17]. Across these studies, radiotherapy consistently improved local control and contributed to favorable overall survival, with five-year overall survival approximately 90% [4]. Collectively, these findings underscore the critical role of radiotherapy in personalized, site-adapted treatment planning for adult PBL.

They also illustrate several important clinical challenges not consistently emphasized in the literature. In Case 1, initial polyclonal flow cytometry findings and nonspecific presentation delayed definitive diagnosis, highlighting the importance of adequate tissue sampling and persistence in cases with high clinical suspicion. Additionally, sacral involvement led to early neurologic and urinary symptoms, an uncommon but clinically significant presentation. In Case 2, multiple incidental findings complicated staging and required careful evaluation to exclude disseminated disease. Furthermore, incomplete metabolic response to first-line therapy necessitated treatment escalation and ultimately higher-dose radiotherapy. This case underscores the importance of response-adapted management and individualized radiotherapy dosing in select patients with residual or refractory disease.

Prognosis and future directions

Prognosis is generally favorable for localized, monostotic PBL but poorer for multifocal disease [16]. Adverse prognostic factors include non-GCB histology, MYC rearrangements, high proliferative index, and delays in diagnosis [12]. Despite excellent outcomes with combined-modality therapy, consensus is limited due to the rarity of PBL, heterogeneity in disease definitions, and inconsistent reporting of molecular features [14]. Future efforts, including prospective registries and PET-adapted treatment strategies, are needed to refine prognostic biomarkers, optimize therapy, and improve long-term outcomes in this rare lymphoma subtype.

## Conclusions

PBL is a rare but highly treatable malignancy that often presents with nonspecific or atypical features, contributing to diagnostic delay. These cases highlight several important clinical considerations. First, atypical presentations, such as sacral involvement with neurologic or urinary symptoms, require a high index of suspicion and may necessitate repeated or more definitive biopsy when initial studies are inconclusive. Second, accurate staging is critical, particularly in the presence of incidental findings that may mimic disseminated disease. From a therapeutic standpoint, combined chemo-immunotherapy and radiotherapy remain the cornerstone of treatment; however, management should be individualized. As demonstrated in our second case, patients with residual or refractory disease may benefit from response-adapted treatment strategies, including escalation of radiotherapy dose beyond standard ranges to optimize local control. Overall, these cases underscore the importance of multidisciplinary evaluation, careful diagnostic interpretation, and flexible, patient-specific treatment planning in achieving favorable outcomes in PBL.
